# The predictive value of neutrophil to lymphocyte ratio for abortion: a systematic review and meta-analysis

**DOI:** 10.3389/fmed.2025.1565979

**Published:** 2025-09-19

**Authors:** Mi Wang, Rui Yue, Jun Xi, Fang Yan

**Affiliations:** ^1^Department of Blood Transfusion, Xi’an No. 3 Hospital, The Affiliated Hospital of Northwest University, Xi’an, China; ^2^Medical Laboratory, XD Group Hospital, Xi’an, China

**Keywords:** neutrophil to lymphocyte ratio, abortion, pregnancy, prognosis, NLR

## Abstract

**Background:**

Abortion usually refers to the loss of pregnancy before viability. Despite a potential link between neutrophil-lymphocyte ratio (NLR) levels and abortion, inconclusive findings remain. This review aimed to comprehensively appraise the predictive utility of NLR levels in abortion, offering a new approach to clarify its potential role as a biomarker.

**Methods:**

PubMed and Cochrane Library were searched for relevant cohort and case–control studies until September 2024. Odds ratio (OR) or weighted mean difference (WMD) with 95% CI of abortion were computed. Subgroup analyses were implemented to clarify potential sources of heterogeneity.

**Results:**

Higher NLR levels were linked to an enhanced risk of abortion as a continuous (WMD, 0.58; 95% CI: 0.29, 0.88) and dichotomous variable (OR, 1.33; 95% CI: 1.03, 1.72). In subgroup analyses, pooled results from studies with NLR cut-off>3, Asia populations, missed abortion, spontaneous abortion, and mean age>30 demonstrated an increased risk of abortion. In continuous NLR for predicting abortion, retrospective study, Europe populations, threatened abortion, recurrent pregnancy loss, and abortion had an enhanced risk of abortion for higher NLR levels as a dichotomous variable.

**Conclusion:**

Pooled analyses demonstrated that higher NLR levels may predict abortion. Further investigations need to determine whether these findings can be generalized to all populations.

**Systematic review registration:**

https://www.crd.york.ac.uk/PROSPERO/view/485726.

## 1 Introduction

According to the WHO definition, abortion refers to the expulsion or removal of a fetus (embryo) weighing < 500 grams (around 22 weeks of gestation). Abortion is usually defined as the loss of pregnancy before viability. With an estimated 23 million abortions worldwide each year, the overall risk of abortion is 15.3% (95% CI, 12.5–18.7%) of all confirmed pregnancies (1). It significantly raises the risk of complications such as coagulation disorders, endometrial damage, and infections, posing great harm to the physical and mental health of pregnant mothers.

The ability of the maternal immune system to fit in the developmental stages of the embryo is crucial for a successful pregnancy. A balanced inflammatory condition is necessary for healthy implantation and tissue remodeling. During embryo implantation, placenta formation, and the first trimester of pregnancy, abortion may be related to placental dysfunction, leading to systemic inflammation in the mother. An excessive maternal inflammatory response is a significant cause of early abortion (2). Many inflammatory cytokines are elevated in the serum of miscarried women (3). However, these markers are not cheap and are not suitable for routine testing. The neutrophil-lymphocyte ratio (NLR), a new index from the complete blood count, reflects the inflammatory state. Due to its availability and widespread use, more articles have stated that NLR is closely associated with the prognosis and incidence of many diseases (4). In recent years, the link between inflammation and abortion has gained more attention. The association between NLR levels and abortion has been extensively investigated. However, the results have been inconsistent (5–24).

There is a growing demand for potent evidence on the NLR level in abortion and its mechanisms. In a recent meta-analysis included 14 articles that Hantoushzadeh et al. revealed that NLR was higher in abortion patients than in healthy controls (25), but subsequent studies have obtained different findings. Çallıoğlu et al. (26) stated that NLR was not greatly distinct between the early pregnancy loss group and the control group, while Humadi et al. (27) showed that NLR was higher in abortion patients. Consequently, this review intended to illustrate the associations between inflammation, NLR levels, and abortion.

## 2 Methods

### 2.1 Literature search

This paper obeyed the PRISMA checklist. The study was prospectively registered in PROSPERO (CRD42023485726). Cochrane Library, PubMed, Embase, and Web of Science were searched until September 2024 for English articles that compared NLR levels between patients with and without abortion. The search terms used were ‘miscarriage’, ‘abortion’, ‘neutrophil’, ‘lymphocyte’, and ‘ratio’. Random combinations of subject terms and free words were utilized to retrieve relevant studies. The specific strategy in Pubmed was ((((“Neutrophils”[Mesh]) OR ((((((((((((((Neutrophil) OR (Leukocytes, Polymorphonuclear)) OR (Leukocyte, Polymorphonuclear)) OR (Polymorphonuclear Leukocyte)) OR (Polymorphonuclear Leukocytes)) OR (Polymorphonuclear Neutrophils)) OR (Neutrophil, Polymorphonuclear)) OR (Polymorphonuclear Neutrophil)) OR (LE Cells)) OR (Cell, LE)) OR (LE Cell)) OR (Neutrophil Band Cells)) OR (Band Cell, Neutrophil)) OR (Neutrophil Band Cell))) AND ((“Lymphocytes”[Mesh]) OR (((((Lymphocyte) OR (Lymphoid Cells)) OR (Cell, Lymphoid)) OR (Cells, Lymphoid)) OR (Lymphoid Cell)))) AND ((abortion) OR (miscarriage))) AND (ratio). Other search strategies are displayed in Supplementary Table S1. Additionally, the reference lists of all eligible studies were manually reviewed. Literature search and evaluation were performed independently by two investigators. Any disagreements were addressed through group discussion with a third investigator to get a final consensus.

### 2.2 Eligible criteria

The inclusion criteria covered: (a) study subjects: pregnant women without internal or obstetric diseases that would disrupt normal pregnancy, like gestational hypertension, infectious diseases, infertility, and gestational diabetes mellitus. (b) NLR = the neutrophil count/the lymphocyte count. (c) Observation population: Women with abortion. (d) Controls: Healthy women with normal pregnancy/delivery. (e) Study design: Observational study.

Articles were excluded for the following reasons (a) review, meeting report, meta-analysis, case report, editorial, comment, letter, note, trial registry record, or protocol. (b) non-human cases, such as animal research. (c) inadequate data on metal concentration. (d) unavailable full text.

### 2.3 Data extraction

Two investigators (Mi W Jun X) performed data extraction independently. Any disagreements were addressed by a third investigator (Fang Y) to make a final decision. The extracted information encompassed the first author, publication year, country, study design, period, abortion type, sample size, age, BMI, gestational age, NLR cut-off, NLR levels, odds ratio (OR), and 95% confidence interval (CI). When continuous variables were depicted as median with range or interquartile range, a validated mathematical method was adopted to calculate the mean ± standard deviation.

### 2.4 Quality assessment

To evaluate the study quality, we employed the Newcastle-Ottawa Scale (NOS), with 7–9 points indicating high quality (28). Two authors (Mi W Jun X) independently appraised each included article, and any disagreements were addressed via discussions with a third author (Fang Y).

### 2.5 Data analysis

Evidence synthesis was conducted utilizing Review Manager 5.4.1. Weighted mean difference (WMD) and OR were adopted for continuous and dichotomous variables. Forest plots were employed to present 95% CIs. Heterogeneity was appraised via Cochran’s Q-test and I^2^. A random-effects model was utilized in case of notable heterogeneity (*p* < 0.05, I^2^ > 50%). To determine the origin of heterogeneity, a subgroup analysis was conducted. Additionally, five subgroup analyses by different types of abortions, the continent of study populations, study type, age, and thresholds of NLR were performed. Publication bias was appraised utilizing funnel plots via Review Manager 5.4.1 and Egger’s tests via Stata 15.1. A *p*-value < 0.05 implied notable publication bias.

## 3 Results

### 3.1 Literature selection and study traits

**Figure 1:**
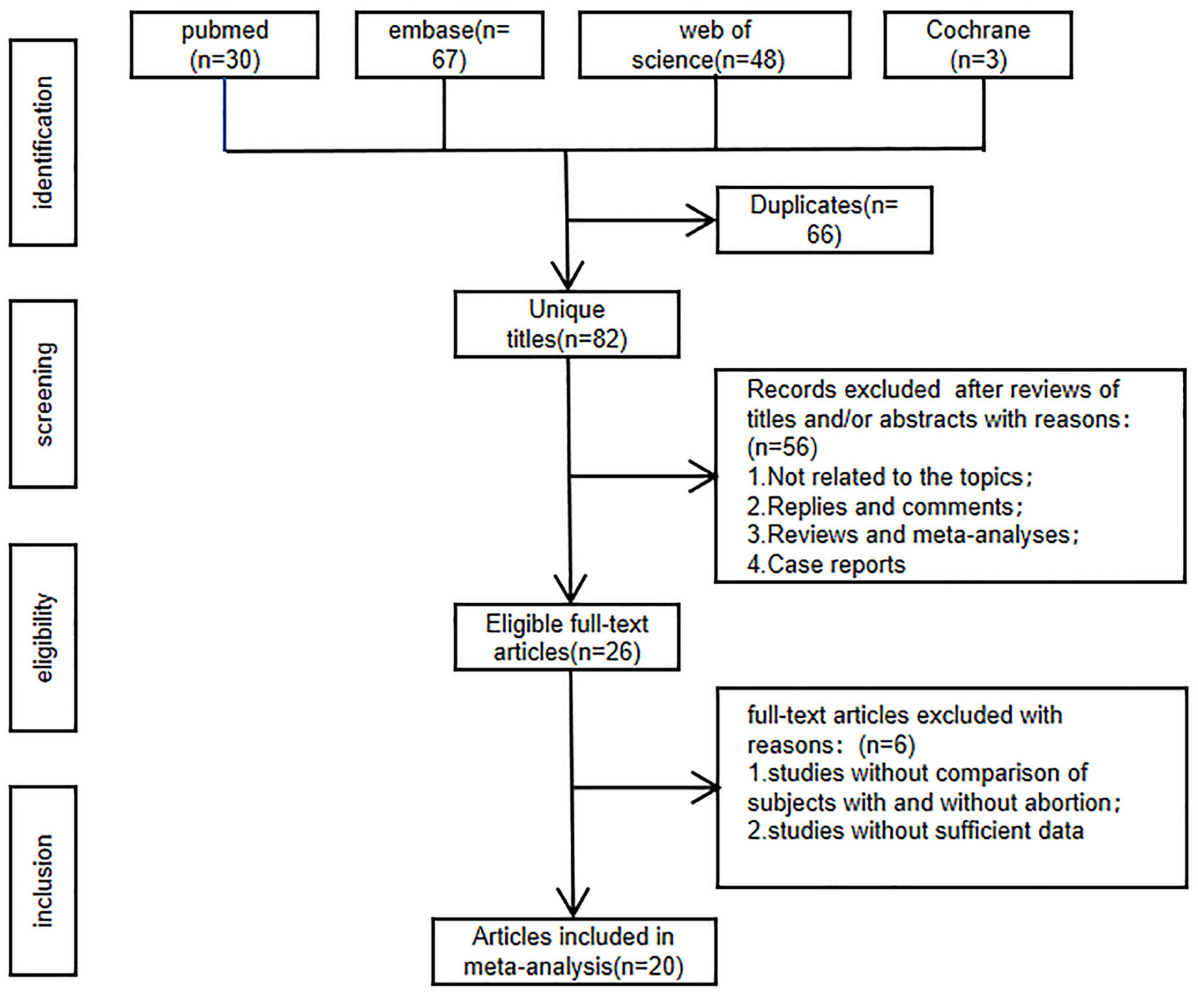
Flowchart of literature screening.

The flowchart of literature screening is presented in Figure 1. 148 articles were found in Web of Science (*n* = 48), PubMed (*n* = 30), Embase (*n* = 67), and Cochrane (*n* = 3). After duplicates were ruled out, the titles and abstracts of 82 documents were scanned. 20 full-text articles with 6,913 patients (3,375 abortion versus 3,538 non-abortion) were finally included. Of these articles, 5 were prospective studies and 15 were retrospective studies. Only studies with quality scores > 6 were considered credible. Table 1 shows the traits and quality scores of the included studies. Quality assessment details are displayed in Supplementary Table S2.

**Table 1:** Baseline study characteristics and methodological assessment.

Authors	Study period	Country	Study design	NLR threshold	Types of abortion	NO.	Age	BMI	Gestational age	Quality
						case/control	case/control	case/control	case/control	score
Yavuz	2018.1–2021.11	Turkey	Retrospective	4.65	Missed abortion	50/50	30.92 ± 5.13/27.85 ± 4.68	NA	11.74 ± 1.56/10.18 ± 1.34	7
Yakıştıran	2019.9–2020.1	Turkey	Retrospective	3.135	Spontaneous abortion	193/164	30.6 ± 6.8/27.8 ± 5.6	NA	7.5 ± 1.2/7.3 ± 1.3	7
Biyik	2015.1–2018.12	Turkey	Retrospective	NA	Missed abortion	40/40	29.27 ± 6.84/28.37 ± 5.13	25.47 ± 5.34/25.89 ± 5.71	54.82 ± 11.54/54.12 ± 12.04(days)	7
Christoforaki	2014.1–2016.7	Greece	Retrospective	NA	Abortion	64/65	NA	NA	NA	6
Wang	2012.6–2018.5	China	Retrospective	2.402	Missed abortion	69/53	28.35 + 3.79/28.60 ± 5.78	22.16 ± 2.25/21.75 ± 2.55	7.43 ± 0.43/7.29 ± 0.43	7
Cimsir	2020.9–2020.12	Turkey	Prospective	4.27	Recurrent pregnancy loss.	44/60	30.5 ± 5.6/29.7 ± 6.0	NA	NA	7
Uckan	2020.1–2022.1	Turkey	Retrospective	2.99	Missed abortion	474/452	28.93 ± 3.99/28.55 ± 4.01	26.62 ± 1.84/26.70 ± 1.99	10.31 ± 0.83/10.49 ± 0.84	7
Aydın	2020.6–2020.11	Turkey	Prospective	2.8182	Threatened abortion	55/55	27.49 ± 5.87/28.25 ± 6.44	24.83 ± 5.45/25.67 ± 5.69	9.53 ± 3.67/10.66 ± 11.63	7
Bas	2012.1–2017.1	Turkey	Retrospective	3.24/3.34	Spontaneous abortion	173/152/245	31.88 ± 6.43/30.87 ± 6.19/30.15 ± 5.62	23.28 ± 1.70/23.56 ± 1.40/23.32 ± 1.65	NA	7
Yazdizadeh	2021.3–2022.3	Iran	Retrospective	NA	Spontaneous abortion	120/120	30.46 ± 4.44 /30.13 ± 4.18	22.52 ± 1.77/22.16 ± 1.73	59.98 ± 4.85/59.28 ± 5.82 (days)	8
Turgut	2020.7–2021.7	Turkey	Retrospective	3.2	Abortion	709/676	30 ± 6/29 ± 6	29 ± 4/28 ± 5	7.6 ± 1.5/9.5 ± 3	7
Soysal	2019.1–2020.12	Turkey	Retrospective	3.94	Threatened abortion	150/150	29.0 ± 6.2/28.2 ± 5.9	28.0 ± 4.1/28.3 ± 3.5	9.1 ± 2.8/9.4 ± 2.5	7
Jiang	2012.1–2018.6	China	Retrospective	3.16	Recurrent pregnancy loss.	133/140	34.1 ± 3.9/33.4 ± 2.9	22.3 ± 2.9/21.8 ± 2.8	NA	7
GORKEM	2018.9–2019.8	Turkey	Prospective	NA	Threatened abortion/ spontaneous abortion	30/30/30	25.5 + 4.2/27.4 + 5.8/25.8 + 3.9	25.89 ± 5.99/24.58 ± 4.90/23.23 ± 3.58	8 ± 1.56/8.29 ± 1.56/8.18 ± 1.17	7
Oğlak	2019.9–2019.12	Turkey	Retrospective	NA	Early pregnancy loss	137/148	23.32 ± 3.26/26.09 ± 3.04	23.12 ± 3.66/23.78 ± 3.82	NA	7
Ata	2018.1–2019.5	Turkey	Retrospective	2.99/2.91	Early pregnancy loss/threatened abortion	100/100/100	27.7 ± 4.7/28.1 ± 4.0/27.1 ± 5.2	NA	10 ± 2.1/11 ± 0.9/10 ± 1.8	8
Sert	2018.1–2021.12	Turkey	Retrospective	2.59	Missed abortion	142/142	28.7 ± 6.9/27.1 ± 5.2	NA	7.9 ± 1.7/7.6 ± 1.2	7
Uysal	2014.4–2014.12	Turkey	Prospective	NA	Missed abortion	90/143	27.2 ± 6.7/26.7 ± 5.7	24 ± 3/24 ± 4	9.6 ± 1.9/9.3 ± 2.4	7
Taskomur	2020.6–2021.8	Turkey	Prospective	2.8083/NA	Threatened abortion/spontaneous abortion	60/60/60	27.38 ± 5.75/29.58 ± 5.52/28.17 ± 6.29	24.76 ± 5.30/26.38 ± 5.95/25.76 ± 5.49	9.69 ± 3.80/10.11 ± 3.51/10.59 ± 11.16	7
Liu	2018.1–2020.12	China	Retrospective	NA	Missed abortion	200/200	27.86 ± 2.93/26.93 ± 2.93	22.14 ± 2.69/21.96 ± 2.15	7.14 ± 1.10/7.14 ± 1.10	7

### 3.2 Meta-analysis

#### 3.2.1 Link between abortion and NLR levels

**Figure 2:**
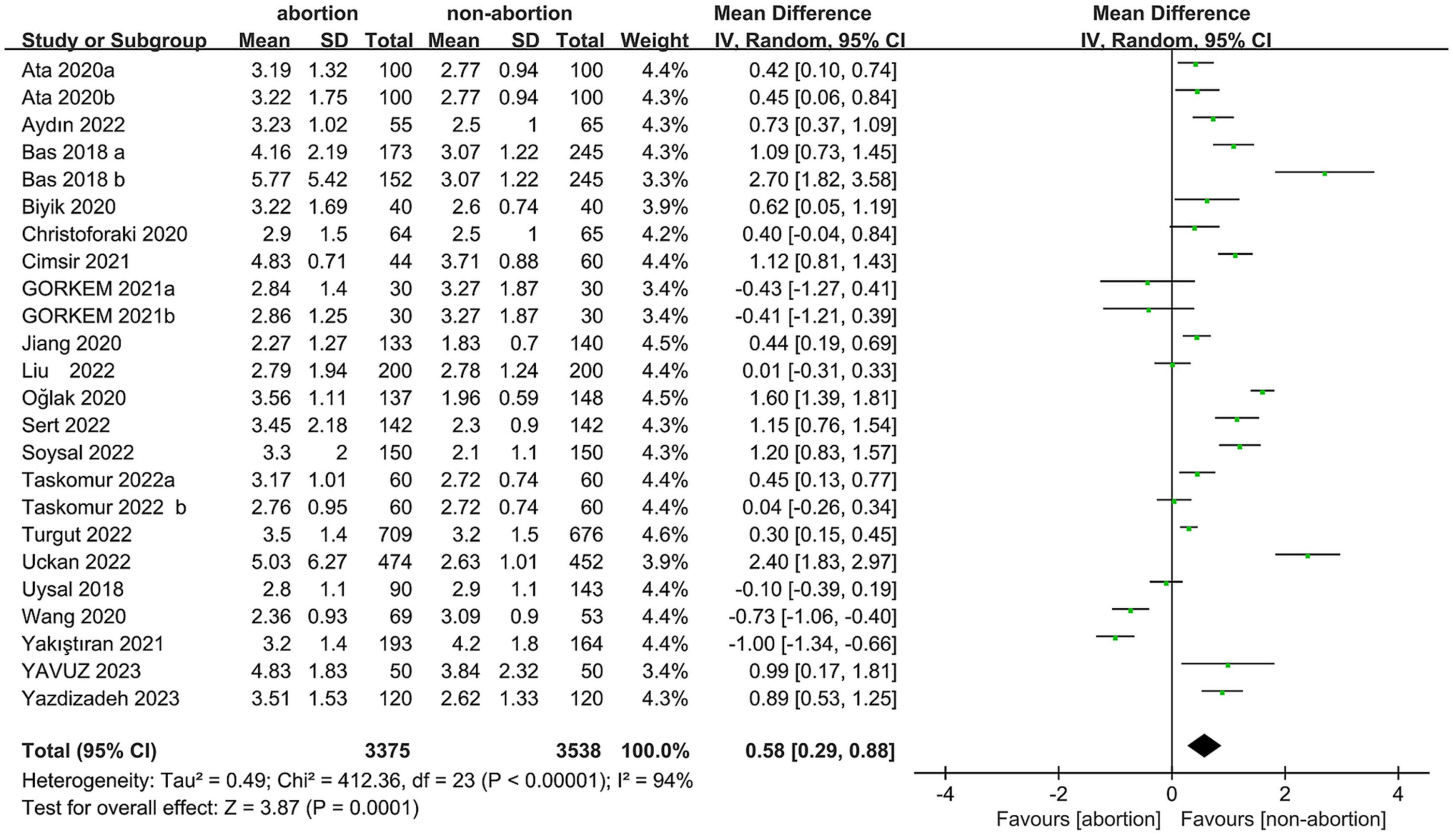
Forest plots of the relationship between abortion and NLR.

**Figure 3:**
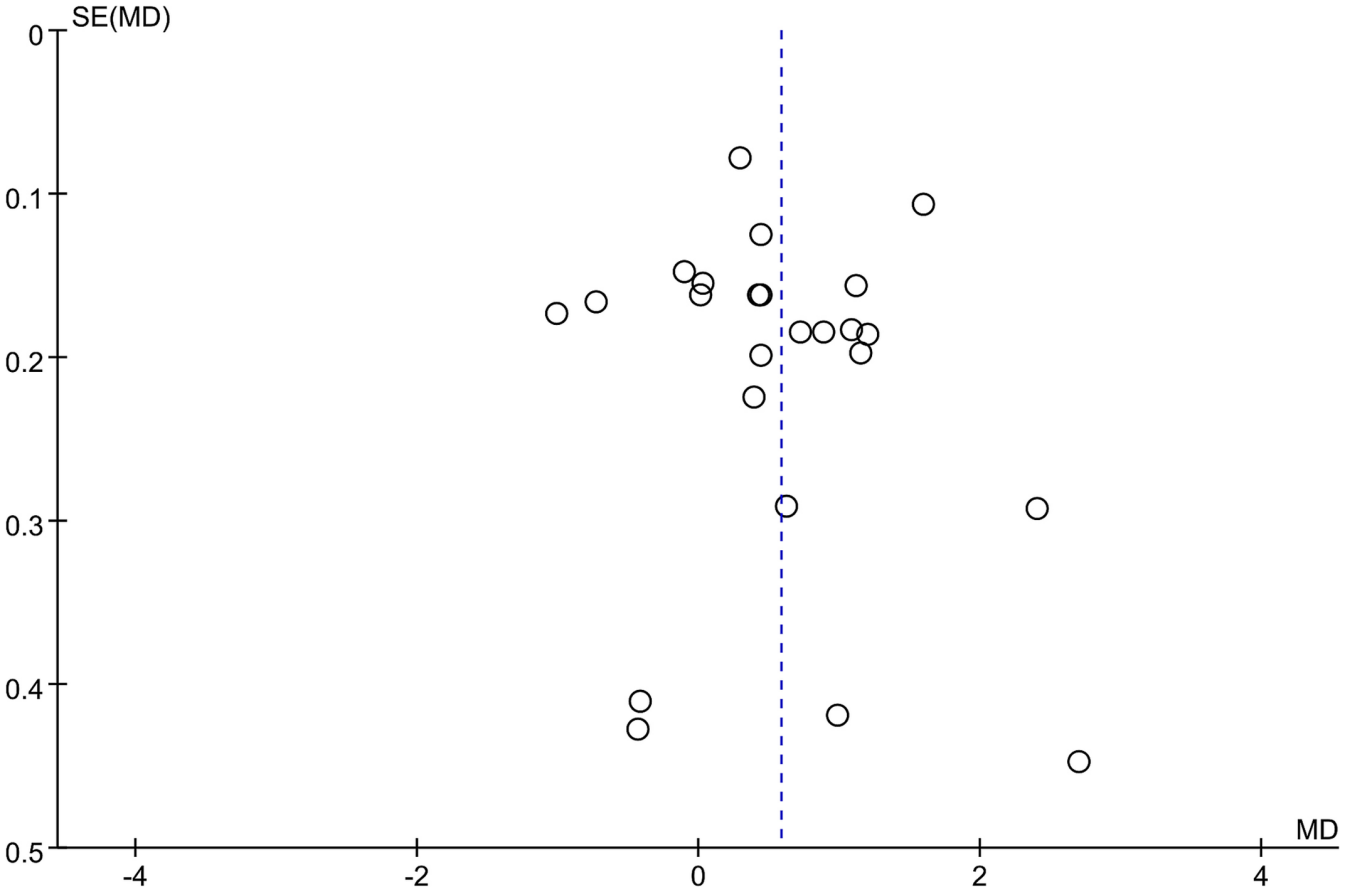
Funnel plots of the relationship between abortion and NLR.

Data were synthesized from 20 studies, containing 6,913 patients (3,375 abortion versus 3,538 non-abortion). Pooled analysis (Figure 2) revealed significantly higher NLR levels in the abortion cohort (WMD: 0.58; 95% CI: 0.29, 0.88; *p* = 0.0001) with notable heterogeneity (I^2^ = 94%, *p* < 0.00001). A funnel plot (Figure 3) noted slight publication bias. But Egger’s test uncovered no publication bias (*p* = 0.700).

#### 3.2.2 The risk of abortion and NLR levels

**Figure 4:**
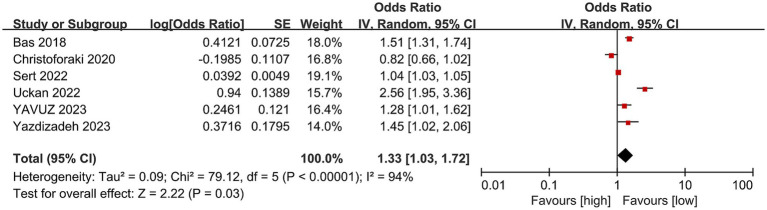
Forest plots of the risk of abortion and NLR.

**Figure 5:**
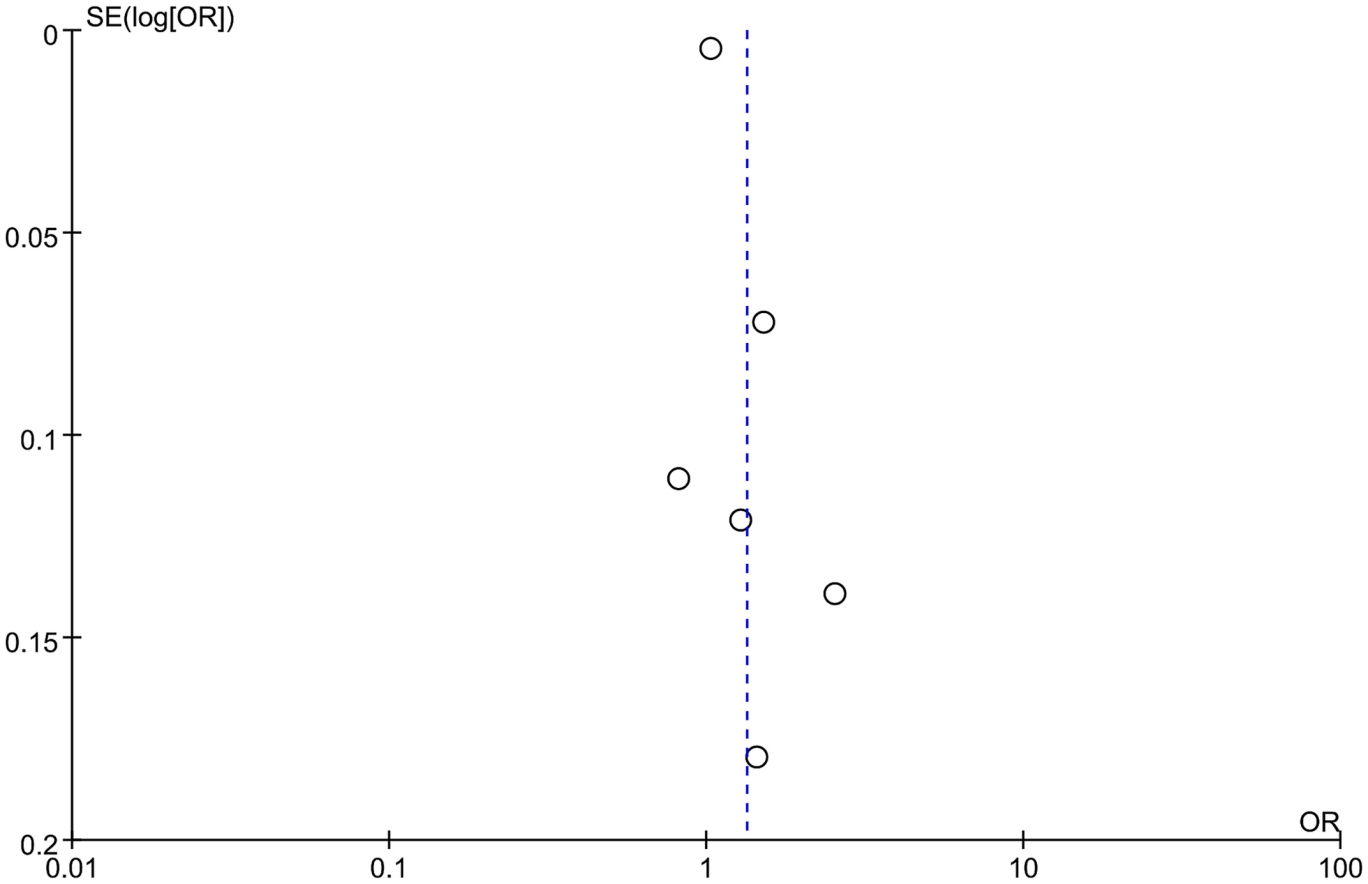
Funnel plots of the risk of abortion and NLR.

6 studies with 2,249 patients (1,175 abortion versus 1,074 non-abortion) were included in the analysis for risk of abortion. Pooled analysis (Figure 4) indicated that pregnant women with high NLR were at greatly higher abortion risk (OR: 1.33; 95% CI: 1.03, 1.72; *p* = 0.03) with marked heterogeneity (I^2^ = 94%, *p* < 0.00001). Both the funnel plot (Figure 5) and Egger’s test (*p* = 0.162) did not showcase publication bias.

### 3.3 Subgroup

Subgroup analyses were done by study design, the NLR threshold, region, types of abortion, and mean age to compare the prevalence of abortion contraceptive utilization across different studies. Subgroup analysis showed that in retrospective articles, abortion patients had higher NLR levels than controls, whereas this result was not observed in prospective articles. Moreover, NLR levels were markedly higher in European cohorts with abortion than in controls, but no significant difference was noted in Asians. Further subgroup analysis unveiled that NLR levels were greatly higher in threatened abortion patients, recurrent pregnancy loss (RPL) patients, and abortion patients than in controls. However, such differences were not observed in the missed abortion, spontaneous abortion, and early pregnancy loss subgroups (Table 2).

**Table 2:** Subgroup analysis.

Subgroup	Risk of abortion				NLR level			
	Study	OR [95%CI]	*p* value	*I* ^2^	Study	MD [95%CI]	*p* value	*I* ^2^
Total	6	1.33 [1.03, 1.72]	0.03	94%	24	0.58 [0.29–0.88]	0.0001	94%
Study design								
Prospective	0	NA	NA	NA	7	0.27 [−0.15–0.68]	0.21	88%
Retrospective	6	1.33 [1.03, 1.72]	0.03	94%	17	0.72 [0.34–1.10]	0.0002	96%
NLR threshold								
*>*3	2	1.43 [1.23, 1.67]	<0.00001	28%	8	0.79 [0.27–1.31]	0.003	95%
≤3	2	1.61 [0.67–3.90]	0.29	98%	7	0.67 [0.08–1.27]	0.03	95%
Region								
Asia	1	1.45 [1.02–2.06]	0.04	NA	4	0.15 [−0.48–0.78]	0.64	94%
Europe	5	1.32 [1.00, 1.74]	0.05	95%	20	0.67 [0.34–1.00]	0.0001	94%
Types of abortion								
Missed abortion	3	1.48 [0.91–2.43]	0.12	96%	7	0.59 [−0.10–1.29]	0.1	95%
Spontaneous abortion	2	1.50 [1.32, 1.71]	<0.00001	0%	6	0.52 [−0.32–1.36]	0.23	96%
Threatened abortion	0	NA	NA	NA	5	0.57 [0.18–0.96]	0.004	78%
Recurrent pregnancy loss	0	NA	NA	NA	2	0.77 [0.11–1.44]	0.02	91%
Early pregnancy loss	0	NA	NA	NA	2	1.02 [−0.14–2.17]	0.08	97%
Abortion	1	0.82 [0.66–1.28]	0.07	NA	2	0.31 [0.17–0.46]	0.0001	0
Mean age								
*>*30	3	1.45 [1.29, 1.62]	<0.00001	0%	7	0.84 [0.17–1.51]	0.01	95%
≤30	2	1.61 [0.67–3.90]	0.29	98%	16	0.49 [0.14–0.85]	0.006	95%

Subgroup analyses by key study characteristics were also performed. Among different NLR cut-offs, an NLR ≥ 3 linked to a prominently higher incidence of abortion. Among different regions, higher NLR was connected to a visibly higher incidence of abortion in Asian patients. Among different types of abortion, higher NLR was connected to a noticeably higher incidence of missed abortion and spontaneous abortion. Among different mean ages, higher NLR was linked to a sensibly higher incidence of abortion when patients’ ages are above 30 (Table 2).

### 3.4 Sensitivity analysis

**Figure 6:**
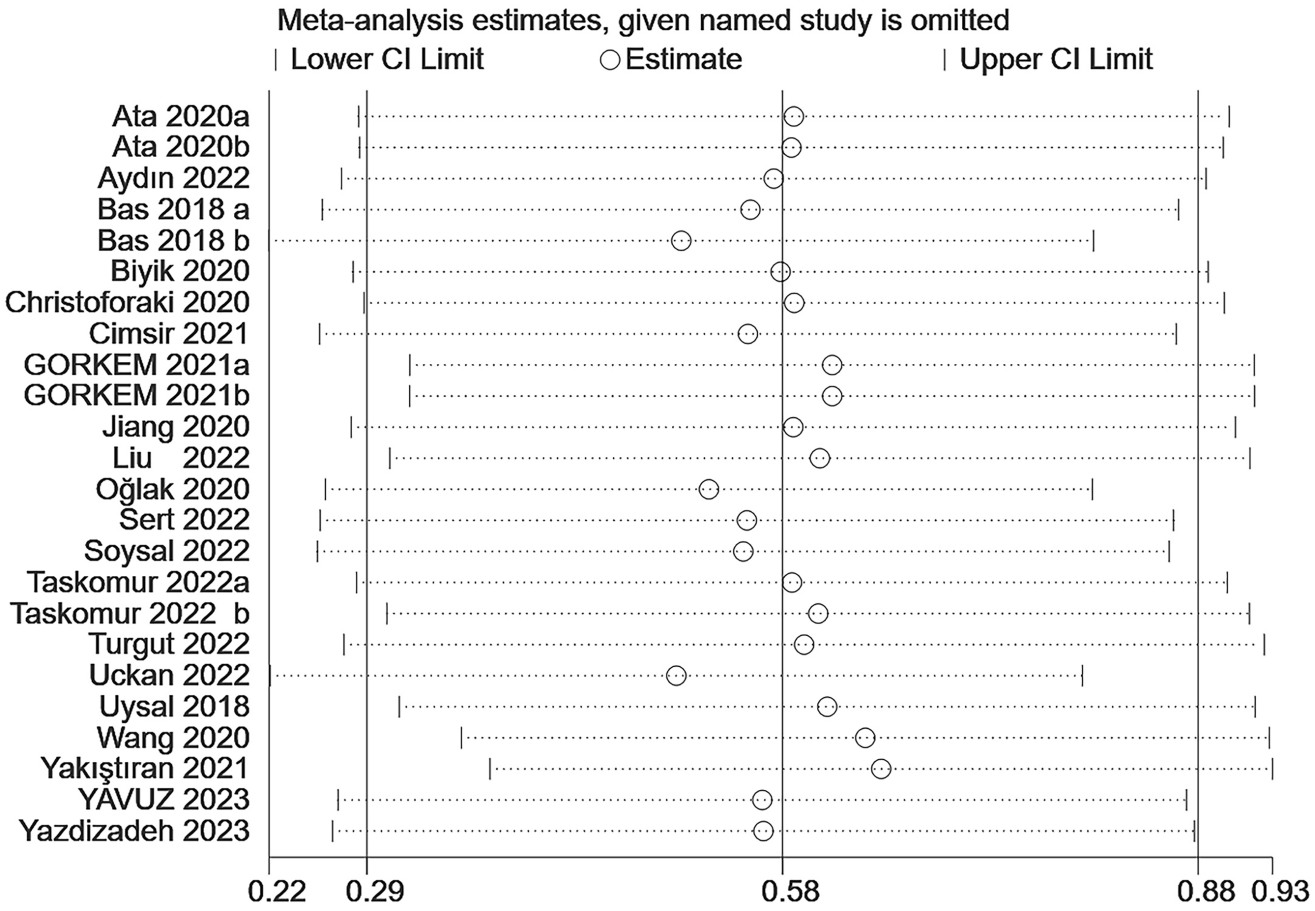
Sensitive analysis of the relationship between abortion and NLR.

**Figure 7:**
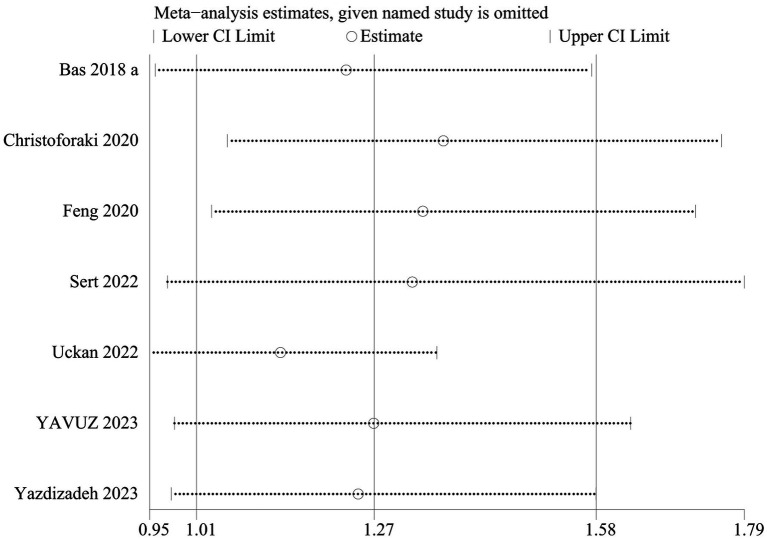
Sensitive analysis of the risk of abortion and NLR.

In the prediction of abortion by NLR, sensitivity analyses evinced that the pooled WMD remained unchanged after exclusion of any single study (Figure 6). However, in the link of NLR with abortion risk, sensitivity analysis unraveled that the removal of three studies (6, 18, 23) altered the overall effect (Figure 7).

## 4 Discussion

This meta-analysis examined the predictive performance of NLR in abortion. In the enrolled 20 articles with 6,913 patients (3,375 abortion versus 3,538 non-abortion), we summarized that NLR could predict abortion.

Subgroup analyses by NLR threshold, regions, types of abortion, and mean age were also performed. Heterogeneity may be due to these factors. Only in NLR cut-off>3, these factors had significant associations with the risk of abortion. A threshold of NLR > 3 is better set to predict abortion. Higher NLR was connected to considerably higher abortion rates in Asia, suggesting that NLR is of greater value in predicting abortion in Asian populations. Further research is needed in other regions. As for different types of abortion, higher NLR was connected to a markedly higher incidence of missed abortion and spontaneous abortion. Neutrophil to lymphocyte ratio may be better at predicting these two types of abortion. Regarding different mean ages, higher NLR was connected to a notably higher incidence of abortion when patients aged above 30. Pregnant women older than 30 are better predicted with NLR. In the relationship between NLR and abortion, only retrospective studies, Europe populations, threatened abortion, RPL, and abortion had significant associations.

Abortion is an extremely distressing incident for couples, leading to diverse psychological consequences. Recent studies have highlighted substantial changes in maternal adaptations and innate immune responses to keep normal pregnancy (29). Under normal pregnancy conditions, there is a marked increase in complete blood count parameters (e.g., white blood cells) and decreases in the proportions of granulocytes, Th-1 lymphocytes, Th-2 lymphocytes, and monocytes (30). Macrophages and monocytes are critical for fetal development since they facilitate extraepithelial trophoblast invasion, spiral dolphin remodeling, and birthing process. In addition, clinical studies have revealed a strong link between inflammation-related parameters and pregnancy complications (31). However, sustained and uncontrolled inflammatory responses have detrimental effects on placental growth, prenatal development, and maternal health (32).

The relationship between inflammation and abortion has garnered more interest in recent years. Experimental research suggests that inflammation is involved in the entire evolution of pregnancy (33, 34). Inflammation and coagulation disorders are crucial in the pathogenesis of abortion, as immunopathological evaluation of abortive material at the site of placenta implantation reveals inflammation and fibrin deposition in the meconium as well as thromboembolism in the meconium vasculature. Normal pregnancies require aseptic inflammation for successful embryo implantation. However, if implantation is uncontrolled and placental growth persists, prenatal development and maternal health may be noted in uterine tissues. Natural Killer cells secrete cytokines that act on the uterus, and activation of vascular endothelial procoagulant initiates ischemia, leading to embryo loss, thrombosis, and inflammation (35). Compared to normal pregnancy, RPL patients have higher levels of cytokines (TNF-a, IFN-*γ*, IL-12, and IL-2) and increased inflammatory response. Nonetheless, the predicting role of NLR in abortion is elusive (35–38). This study illustrated that higher NLR levels predicted a higher risk of abortion.

There are certain limitations. First, the number of retrospective studies is numerous with low quality. Second, marked heterogeneity was noted. Thus, sensitivity and subgroup analyses were conducted to judge the result stability. There are also strengths. First, this is the meta-analysis with the largest sample size to determine the link between NLR and abortion. Second, the estimates based on WMD and OR data were pooled, which made the results more reliable. Third, data were pooled to evaluate the predictive utility of NLR in abortion. Further prospective studies are needed.

## 5 Conclusion

Pooled analyses demonstrated that NLR may serve as a potential predictor of abortion. The risk of abortion increases with a higher NLR. The number of retrospective studies and studies conducted in Europe is relatively large. Due to heterogeneity, further studies need to determine whether the findings can be generalized to all populations.

## Data Availability

The original contributions presented in the study are included in the article/Supplementary material, further inquiries can be directed to the corresponding author.
