# Supplementary material for: The predictive value of neutrophil to lymphocyte ratio for abortion: a systematic review and meta-analysis
Source: Front Med (Lausanne). 2025 Sep 19;12:1565979. doi: 10.3389/fmed.2025.1565979 (PMC12491316; doi:10.3389/fmed.2025.1565979)
Supplement: Supplementary file 1 [file Table_1.docx]

**Table S1.** The detailed search strategy

Pubmed: 30

(((("Neutrophils"[Mesh]) OR ((((((((((((((Neutrophil) OR (Leukocytes, Polymorphonuclear)) OR (Leukocyte, Polymorphonuclear)) OR (Polymorphonuclear Leukocyte)) OR (Polymorphonuclear Leukocytes)) OR (Polymorphonuclear Neutrophils)) OR (Neutrophil, Polymorphonuclear)) OR (Polymorphonuclear Neutrophil)) OR (LE Cells)) OR (Cell, LE)) OR (LE Cell)) OR (Neutrophil Band Cells)) OR (Band Cell, Neutrophil)) OR (Neutrophil Band Cell))) AND (("Lymphocytes"[Mesh]) OR (((((Lymphocyte) OR (Lymphoid Cells)) OR (Cell, Lymphoid)) OR (Cells, Lymphoid)) OR (Lymphoid Cell)))) AND ((abortion) OR (miscarriage))) AND (ratio)

Embase: 67


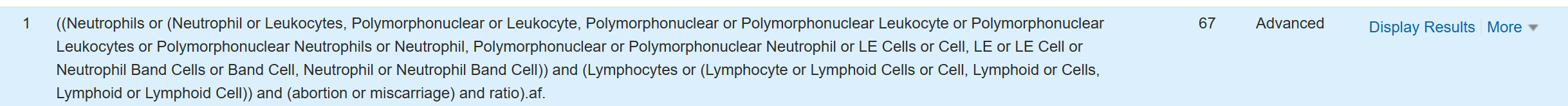


Cochrane: 3


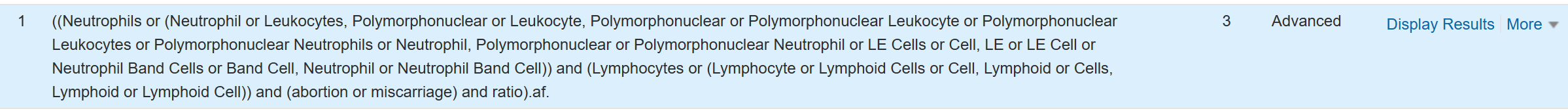


WOS: 48

((((Neutrophils) OR ((((((((((((((Neutrophil) OR (Leukocytes, Polymorphonuclear)) OR (Leukocyte, Polymorphonuclear)) OR (Polymorphonuclear Leukocyte)) OR (Polymorphonuclear Leukocytes)) OR (Polymorphonuclear Neutrophils)) OR (Neutrophil, Polymorphonuclear)) OR (Polymorphonuclear Neutrophil)) OR (LE Cells)) OR (Cell, LE)) OR (LE Cell)) OR (Neutrophil Band Cells)) OR (Band Cell, Neutrophil)) OR (Neutrophil Band Cell))) AND ((Lymphocytes) OR (((((Lymphocyte) OR (Lymphoid Cells)) OR (Cell, Lymphoid)) OR (Cells, Lymphoid)) OR (Lymphoid Cell)))) AND ((abortion) OR (miscarriage))) AND (ratio) (Topic) and Preprint Citation Index (Exclude – Database)

**Table S2.** Quality evaluation of the eligible studies with Newcastle–Ottawa scale.

| Study | Selection | | | | Comparability | | Exposure | | |
| --- | --- | --- | --- | --- | --- | --- | --- | --- | --- |
|  | Adequate definition | Representative-ness | Selection of  Controls | Definition of  Controls | Comparability on most important factors | Comparability on other risk factors | Ascertainment of exposure | Same method of Assessment for cases and controls | Non-response rate |
| Onur YAVUZ 2023 | * | * | * | * | - | - | * | * | * |
| Betül Yakıştıran 2021 | - | * | * | * | * | - | * | * | * |
| Ismail Biyik 2020 | * | * | * | * | - | - | * | * | * |
| Viktoria Christoforaki2019 | - | * | * | * | - | - | * | * | * |
| Qingjie Wang 2020 | * | * | * | * | - | - | * | * | * |
| Meral Tugba Cimsir 2021 | * | * | * | * | - | - | * | * | * |
| Kazim Uckan 2022 | * | * | * | * | - | - | * | * | * |
| Sakine Merve Aydın 2022 | * | * | * | * | - | - | * | * | * |
| Funda Yildirim Bas 2018 | * | * | * | * | - | - | * | * | * |
| Maryam Yazdizadeh 2023 | * | * | * | * | * | - | * | * | * |
| Ezgi Turgut 2022 | * | * | * | * | - | - | * | * | * |
| Cenk Soysal 2022 | * | * | * | * | - | - | * | * | * |
| Sushi Jiang 2020rpl | * | * | * | * | - | - | * | * | * |
| Umit GORKEM 2021 | * | * | * | * | - | - | * | * | * |
| Süleyman Cemil Oğlak 2020 | * | * | * | * | - | - | * | * | * |
| Nahit Ata 2020 | * | * | * | * | - | - | * | * | * |
| Zekiye Soykan Sert 2022 | * | * | * | * | - | - | * | * | * |
| Gülsüm Uysal 2018 | * | * | * | * | - | - | * | * | * |
| Aysun Tekeli Taskomur 2022 | * | * | * | * | - | - | * | * | * |
| Liu 2022 | * | * | * | * | - | - | * | * | * |
| *indicates criterion met; - indicates significant of criterion not met. | | | | | | | | | |
